# The long-term outcomes and health-related quality of life of patients following blunt thoracic injury: a narrative literature review

**DOI:** 10.1186/s13049-018-0535-9

**Published:** 2018-08-17

**Authors:** Edward Baker, Andreas Xyrichis, Christine Norton, Philip Hopkins, Geraldine Lee

**Affiliations:** 1 Florence Nightingale Faculty of Nursing, Midwifery and Palliative Care, King’s College London, James Clerk Maxwell Building, 57 Waterloo Road, London, SE1 8WA UK; 2 Emergency Department, King’s College Hospital NHS Foundation Trust, Denmark Hill, London, SE5 9RS UK; 3 Department of Intensive Care Medicine, King’s College Hospital NHS Foundation Trust, Denmark Hill, London, SE5 9RS UK

**Keywords:** Trauma, Rib fractures, Chest trauma, Quality of life, Patient reported outcomes, Injury, Morbidity

## Abstract

**Background:**

Major Trauma remains a leading cause of mortality and morbidity worldwide. Blunt Thoracic Injury (BTI) accounts for > 15% of United Kingdom (UK) trauma admissions and is consistently associated with respiratory related complications that include pneumonia and respiratory failure. Despite this, it is unclear in current clinical practice how BTI impacts on the recovering trauma patients after discharge from hospital. This study aimed to investigate the state of knowledge on the impact of BTI on the long-term outcomes and health-related quality of life (HRQoL).

**Methods:**

Data were sourced from Ovid MEDLINE, Ovid EMBASE, CINAHL and Science Direct using a pre-defined systematic search strategy. A subsequent hand search of key references was used to identify potentially missed studies. Abstracts were screened for eligibility and inclusion. Fifteen studies met the eligibility criteria and were critically appraised. Data were extracted, analysed and synthesised in categories and sub-categories following a narrative approach.

**Results:**

Three major themes were identified from the 15 studies included in this review: (i) physical impact of BTI, (ii) psychological impact of BTI and (iii) socio-economic impact of BTI. The bulk of the available data focused on the physical impact where further sub-themes included: (i) physical functioning, (ii) ongoing unresolved pain, (iii) reduced respiratory function, (iv) thoracic structural integrity. Although there was a substantial difference in the length and method of follow up, there remains a general trend towards physical symptoms improving over time, particularly over the first six months after injury. Despite this, where sequelae continued at six months it remained likely that these would also be present at two years after injury.

**Conclusion:**

The literature review demonstrated that BTI is associated with substantial sequelae that impacts on all aspects of daily functioning. Despite this there remains a paucity of data relating to long term outcomes in the BTI population, especially relating to psychological and socio-economic impact. There is also little consensus on the measures, tools and time-frames used to measure outcomes and HRQoL in this population. The full impact of BTI on this population needs further exploration.

**Electronic supplementary material:**

The online version of this article (10.1186/s13049-018-0535-9) contains supplementary material, which is available to authorized users.

## Background

Major trauma continues to be a leading cause of death for both men and women under the age of 44 years in high-income countries [1, 2]. Blunt Thoracic Injury (BTI) is characterised by injury that does not involve opening of the chest wall and therefore the severity can vary from minor haematoma to significant injury that compromises thoracic structural integrity [2, 3]. Isolated BTI accounts for over 15% of UK trauma admissions and is consistently associated with a high level of respiratory-related complications such as pneumonia, respiratory failure and subsequent pulmonary embolism [2, 4–6].

It is often challenging for healthcare professionals (HCP) to comprehend the impact of disease on patients’ daily lives [7, 8]. Patient Reported Outcome Measures (PROMs) have been developed to meet these challenges and reduce the gap between clinicians’ and patients’ understanding of these, therefore focusing care on patients’ preference and needs [8]. Despite evidence suggesting that PROMs can improve communication between HCPs and patients whilst promoting patient autonomy and improving patient satisfaction, their use in trauma care requires further development [9–12]. Furthermore, there are very limited trauma specific PROMs available and currently insufficient evidence to fully understand the effectiveness of generic measures in the trauma population [12, 13]. Whilst further investigation of PROMs in trauma care is needed, it is important to investigate how these measures are currently utilised and presented in the literature.

In the general major trauma population, physical injury has been shown to impact on all aspects of quality of life [14–21]. This has resulted in negative changes to physical functioning including ability to sleep, changes in psycho-sexual function and effects on employment, financial status and ability to return to work [20, 22–26]. Despite these documented sequelae, it is unclear in current clinical practice how BTI specifically impacts on the recovering trauma patient and their health-related quality of life (HRQoL) after discharge from hospital [27].

HRQoL is a challenging term to define due to its multi-dimensional nature [28]. It is broadly agreed that HRQoL is the functional effect of a medical problem and its consequent treatment upon the individual and their daily life, involving their perceptions of physical, mental, emotional and social functioning [29]. In those with blunt thoracic injury, the identification of potential variables that have an impact on HRQoL can help determine the effectiveness of interventions, such as analgesic modes or evidence-based care pathways, for patients with blunt thoracic injury [12, 30–32].

### Aim of the study

The aim of this literature review was to identify and synthesise the current state of evidence related to the long-term outcomes that are associated with BTI. Specific objectives of this review include:

- Identify the impact of BTI on the long-term functional outcomes of major trauma patients;
- Explore which outcome measures have been used in previous research to measure these long-term functional outcomes; and
- Identify which physical, psychological and socio-economic sequelae impact on the long-term functional outcomes and HRQoL of patients with BTI.

## Method

A narrative review methodology was used to identify and synthesise the literature. This review used a recognised method of planning the search, critically selecting relevant papers, identifying themes through in-depth analysis and then applying these findings in the context of this study [33].

Five databases were used to identify the studies included in this literature review: Ovid Medline, Ovid EMBASE, Cumulative Index to Nursing and Allied Health (CINAHL) and Science Direct. All databases were searched from inception to September 2017. The search was developed using a combination of search terms: ‘trauma’, ‘injury’, ‘patient reported outcomes’, ‘long term outcomes’ and ‘impact’. Key papers were also hand searched for additional unidentified studies. At this stage all levels of evidence were deemed eligble for inclusion in this review.

Data extraction from each study was standardised using a predetermined table and focused on: research design, sample characteristics and size, outcome measures and tools, setting and research methods. As part of the data extraction process, outcome measures used in each study were identified. Included studies were then critically appraised for quality using the relevant assessment tool from the Critical Appraisal Skills Programme (CASP) [34]. The variability in the validity and reliability of the outcome measures and the method of measuring the outcomes are considered in the critical appraisal of the research studies.

## Results

**Fig. 1:**
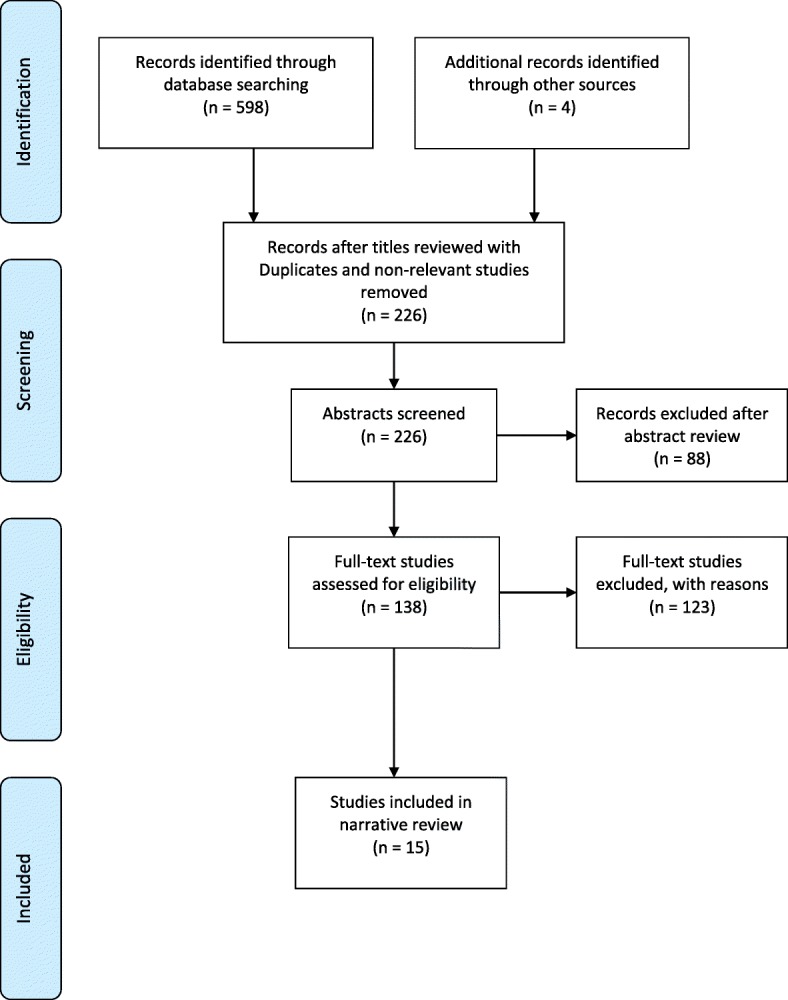
PRISMA Flow Chart summarising study selection [70]

The combined results of the database searches identified 598 published studies. The initial screening by title and study type resulted in the exclusion of 376 studies due to irrelevance to the topic and duplication. The remaining 226 abstracts were obtained for further assessment of relevance and a further 88 studies were excluded for not meeting the eligibility criteria. The review of titles and abstracts was undertaken by the primary author (EB) and discussed at team meetings. Studies published in English that were primary quantitative or qualitative research reports investigating long-term outcomes and HRQoL measures after BTI were included. The remaining 138 studies were reviewed in full by EB resulting in the exclusion of a further 124 studies. Justification for the 211 studies which were excluded are as follows: focused on a population that was less than 16 years of age (*n* = 1), focused on outcomes in the non-BTI trauma population (*n* = 126), focused on acute care outcomes (*n* = 11), non-research articles (*n* = 71) or where participants did not require admission to hospital (*n* = 2). Any uncertainty surrounding article inclusion/exclusion was discussed and an agreement reached based on the eligibility criteria. This resulted in 15 studies that met all eligibility criteria for the review and were included in the data analysis and synthesis (Fig. 1**).**

Table 1 presents a summary of the critical appraisal process. The 15 studies were compared and then synthesised to form emergent and final themes.

**Table 1:** Critical Appraisal Summary Table

Quantitative Observational study: CASP Appraisal Tool										
Author	Clear Focus	Recruitment	Outcome measures	Exposure measured	Confounding factors	Confounding factors-controlled	Was follow up complete	Results	Precise results	Local application
Marasco et al. (2015)	+	+	+	+	+	+	+	+	+	+
Gordy et al. (2014)	+	+	+	+	?	?	+	+	+	+
Marasco et al. (2013)	+	+	+	+	+	+	+	+	+	+
Daoust et al. (2013)	+	+	+	+	?	+	+	+	+	?
Fabricant et al. (2013)	+	+	?	+	+	+	+	+	+	+
Bille et al. (2013)	+	?	+	+	–	–	–	+	?	+
Shelat et al. (2012)	+	+	–	?	?	?	+	?	?	?
Amital et al. (2009)	+	–	+	+	?	?	–	+	?	+
Mayberry et al. (2009)	+	+	+	+	?	?	–	?	?	+
Leone et al. (2008)	+	+	?	+	?	?	+	+	+	+
Kerr-Valentic et al. (2003)	+	+	+	+	?	?	+	+	+	+
Mouton et al. (1997)	+	?	+	+	+	+	–	+	?	+
Beal & Oreskovich (1985)	+	+	?	?	–	–	–	–	?	+
Landercasper et al. (1984)	+	+	+	+	–	–	–	–	?	+
Qualitative Study: CASP Appraisal Tool										
Author	Clear aims	Appropriate Methodology	Appropriate design	Appropriate strategy	Data Collection	Participant relationship	Ethical considerations	Data analysis	Findings	Value of research
Claydon et al. (2017)	+	+	+	+	+	?	+	+	+	+
	Key:	Quality Criterion met +		Partially met ?		Not met -				

The 15 studies included a total of 1923 participants from eight different countries. Of the studies included, 13 were prospective observational studies (*n* = 12 single centre, n = 1 multi-centre), one was a Randomised Controlled Trial (RCT) and one study used a qualitative interview design. In nine of the studies, patient outcomes were measured during a single post-discharge follow-up undertaken between 50 days to 12 years after injury. Four studies undertook repeated follow-up of participants through a pre-defined time frame between one-month and two years following BTI. In the quantitative studies included, there was substantial variance in the sample sizes with eight studies recruiting less than 50 participants (range: 10–734). Table 2 reviews the injury characteristics and demographics of participants included.

**Table 2:** Patient demographics and Injury Characteristics

Author / Sample size	Age Mean (±SD)	Sex (% male)	Injury Characteristics/Sub-groups	No. of thoracic fractures (mean (±SD)	ISS (mean (±SD))	Mortality:
Claydon et al. (2017) (*n* = 15)	Range: 41–63	73.3%	Not reported	Not reported	Not reported	Not reported
Marasco et al. (2015) (*n* = 397)	53.9 (±18.8)	75.1% (*n* = 298)	Group 1: Isolated Chest Injury: 54.4% (*n* = 216) Group 2: Associated ETI: 45.6% (*n* = 181) Presence of Flail Segment (in total sample): 53.1% (*n* = 211)	Not Reported	Group 1: 16.0 (±7.3) Group 2: 30.1 (±11.6) p < 0.001	Group 1: 6.8% (n = 15) Group 2: 14% (n = 26) *p* = 0.02
Gordy et al. (2014) (n = 203)	Not reported	71.4% (*n* = 145)	22% (*n* = 44) = bilateral rib fractures 7% (n = 15) had flail chest 45% (*n* = 92) had ETI	5.4 (range: 1–29)	20 (range: 1–59)	2% (n = 4) died due to complications of the injuries
Daoust et al. (2013) (*n* = 734)	54 (±17)	63.5% (*n* = 466)	≥ 1 rib # = 32.3% (*n* = 237) ≥ 2 rib # = 16.9% (*n* = 124)	Not Reported	Not Reported	Not Measured or Reported
Marasco et al. (2013) (n = 46)	Group 1: 57.8 (±17.1) Group 2: 59.3 (±16.4) *p* = 0.72	Group 1: 43.4% (n = 20) Group 2: 43.4% (*n* = 20) *p* = 1.0	Group 1: Operative Fixation of rib # Group 2: Conservative management of rib #	Group 1: 11.0 (±3.1) Group2: 11.3 (±4.7) *p* = 0.79	Group 1: 35.0 (±11.4) Group2: 30.0 (±6.3) *p* = 0.13	One death prior to discharge from hospital.
Bille et al. (2013) (n = 10)	Median age: 58 years (range: 21–80)	50% (n = 5)	2–3 rib fractures: n = 4 4–6 rib fractures: n = 3 single fracture mal-union: *n* = 3 100% received rib fixation	Median (range) Synthes Prosthesis: 3 (1–4) Stratos Prosthesis: 6 (1–6)	Not reported	No patients died during follow-up period
Sheelat et al. (2012) (*n* = 102)	Median age: 56 (range: 19–84)	71.6% (n = 73)	45.1% (n = 46) had haemothorax or pneumothorax 66.7% (*n* = 68) required chest tube *n* = 16 required ventilatory support on intensive care	50.9% (*n* = 52) = ≤3# 42.2% (*n* = 43) = > 3# 6.9% (*n* = 7) = Flail Chest	20.1 61.8% (*n* = 63) had ISS > 15	Not reported
Fabricant et al. (2013) (n = 203)	Not reported	71.4% (n = 145)	50% = posterior Fractures 26% = Lateral Fractures 24% = anterior Fractures	5.4 (range: 1–29)	20 (range: 1–59)	2% (n = 4) died due to complications of the injuries
Mayberry et al. (2009) (n = 15)	Mean age: 60.6 (range: 30–91)	Not reported	Group 1: Isolated BTI: 17.5% (n = 7) Group 2: ETI: 82.5% (*n* = 33)	Not measured or reported	30 (±12)	Not reported
Amital et al. (2009) (*n* = 13)	44.6 (±13 years)	69.2% (n = 9)	100% = lung contusions 23% (n = 3) = sternal # 76.9 (n = 10) = Haemo/pneumothorax	84.6% (n = 11) had > 3 rib #	Not Reported	Not Reported
Leone et al. (2008) (*n* = 55)	Mean age: 35 (range 22–45)	78% (n = 43)	100% required admission to ICU after BTI	Not Reported	Not Reported	n = 14 died on ICU n = 5 died after ICU d/c
Kerr-Valentic et al. (2003) (n = 40)	52 (±18) (range: 18–100)	67.5% (*n* = 27)	Isolated BTI: 57.5% (n = 23) ETI: 42.5% (*n* = 17) Divided into four groups: Group 1 = ≤2 rib fractures with no ETI (n = 8) Group 2 = ≤2 rib fractures with ETI (n = 6) Group 3 = ≥3 rib fractures with no ETI (n = 9) Group 4 = ≥3 rib fractures with ETI (*n* = 9)	2.7 (±1.7) ≤ 2 rib fractures: 45% (n = 18) ≥ 3 rib fractures: 55% (n = 22)	Not reported	30-day mortality: 2.5% (n = 1)
Mouton et al. (1997) (*n* = 23)	50 (range: 38–53)	Not reported	Flail Chest: (n = 23) 100% received fib fixation	Not reported	Not reported	30-day survival: 91.3% (n = 21)
Beal and Oreskovich (1985) (n = 20)	Isolated BTI: 46.6 (range: 32–67) ETI: 38 (range: 25–55)	Not reported	Group 1: Isolated flail chest (n = 11) Group 2: Flail chest + ETI (n = 9)	Isolated BTI: 9.3 (range: 4–15) ETI: 7.5 (range: 1–16)	Isolated BTI: 20.75 ETI: 33	Not reported
Landercasper et al. (1984) (*n* = 62)	52 (range: 7–87)	64.2% (n = 46)	Flail Chest: (n = 62) 87% (*n* = 54) + ETI 92% (*n* = 57) had one or more concomitant thoracic injuries	Not reported	Not reported	Patients ≤65 years = 7% Patients ≥66 years = 29%

*BTI* Blunt Thoracic Injury *ETI* Extra-Thoracic Injury *ISS* Injury Severity Score

Three overarching themes were identified: i) Physical impact of BTI; ii) Psychological impact of BTI; and, iii) Socio-economic impact of BTI. There are relationships and inter-dependence between each of the three themes and each theme links directly to HRQoL. Table 3 presents a summary of the key findings from studies included in the review.

**Table 3:** Summary of Key Results

Reference/ Sample Size/ Country	Country of origin	Study Design	Duration of follow up	Data Analysis	Key outcomes measured	Summary of Key Results
Claydon et al. (2017) (n = 14)	UK Single Centre	Qualitative interview study	Interviews between 4 and 9-months after injury	Interpretive Phenomenological Analysis	Exploration of the experience and challenges associated with recovering after BTI	*Struggling with breathing and pain* were identified as key symptoms Initially, participants reported ‘feeling scared they may not survive’. These symptoms progressively improved but feeling ‘out of puff’ often continued. Many felt *life was on hold*. ‘Healing was considered a natural process’ which people couldn’t control producing frustration while awaiting for healing. Many thought they would not completely recover and eventually accepted functional limitations. *Many felt lucky to be alive*. All participants reported ‘feeling lucky to be alive’ and was related to an alteration in outlook toward making the most out of life.
Marasco et al. (2015) (n = 397)	Australia Single Centre	Prospective Observational study	24-months	Univariate and Multivariate Analysis	Glasgow Outcome Scale SF-12 at 6,12,24 months VAS pain Score	Over the 2 year follow up major trauma patients with multiple rib fractures exhibited substantially *reduced HRQoL* when compared to the published Australian normative data at all time points. *Return to work* rates were poor with only 71% of those working prior to the injury returning to any work within the 2-year follow-up.
Gordy et al. (2014) (n = 203)	USA Single Centre	Prospective Observational study	6-months	Univariate and Multivariate Analysis	SF-36 Health survey McGill Pain Questionnaire Present Pain Intensity Scales	The incidence of chronic *pain* was 22% and *disability* was 53%. Acute PPI predicted Chronic pain. Associated injuries, bilateral rib fractures, injury severity score, and number of rib fractures were not predictive of chronic pain. No acute injury characteristics were predictive of disability.
Marasco et al. (2013) (n = 46)	Australia Single Centre	Randomised Controlled Trial	6-months	Univariate and Multivariate Analysis	SF-36 Health Survey at 6 months Spirometry Results at 3 months 3D CT results at 3 months	Participants receiving operative fixation had significantly shorter ICU *length of stay* and had reduced requirements for *NIV post extubation*. No differences in spirometry results at 3 months No difference in HRQoL at 6 months
Daoust et al. (2013) (n = 734)	Canada Multi-centre	Prospective Observational Study	Follow-up at 1-month and 3-months	Univariate/Multivariate analysis and trajectory modelling	Pain Score (Range: 0–10)	18.2% of participants reported experiencing substantial *pain* at 90 days after injury and identified a pain trajectory with similar characteristics. Multivariate modelling identified 2 or more rib fractures, smoking and initial oxygen saturations less than 95% to be predictors of on-going pain at 90 days after injury
Bille et al. (2013) (n = 10)	UK Single Centre	Prospective observational study	Mean follow up 14 months (range: 8–23.5)	Univariate Analysis	Pain Visual Analogue Scale (VAS) QoL EORTC QLQ-C30	Seven patients scored the *pain* as 0, one as 1 (mild), one as 4 (moderate) and one as 8 (severe). Only two patients where using PRN analgesics. Only one patient presents severe limitation in his daily life, scoring his QOL as poor.
Fabricant et al. (2013) (n = 203)	USA Single Centre	Prospective observational study	2-month	Univariate and Multivariate Analysis	McGill Pain Questionnaire: Pain Rating Index (PRI) and Present Pain Intensity (PPI) scale	59% (n = 110) patients had prolonged chest wall *pain* and 76% (*n* = 142) had prolonged *disability*. In patients with isolated rib fractures, *n* = 67 (64%) had prolonged chest wall pain and *n* = 69 (66%) had prolonged disability. MPQ PPI was predictive of prolonged pain (odds ratio [OR], 1.8; 95% confidence interval [CI], 1.4 to 2.5), and prolonged disability (OR, 2.2; 95% CI, 1.5 to 3.4). A significant associated injury was predictive of prolonged disability (OR, 5.9; 95% CI, 1.4 to 29).
Shelat et al. (2012) (n = 102)	Singapore Single Centre	Prospective observational study	Single episode of follow-up 1 year after injury	Univariate Analysis	Unvalidated assessment of quality of life	22.5% (n = 23) complained of *chronic pain*. Of these, 26% (n = 6) regularly used analgesic agents, 35% (n = 8) complained of impaired *work life* and 13% (*n* = 3) complained of impaired personal *QoL*. Chronic pain was not related to age, number of rib fractures, flail chest, haemothorax and/or pneumothorax, chest tube insertion or Injury Severity Score (ISS).
Amital et al. (2009) (n = 13)	Israel Single Centre	Prospective observational study	Single episode of follow-up	Univariate Analysis	Lung function Tests	*Lung function* test results: Mean forced expiratory volume in the first second was 85 (±13), residual volume was 143 ± 33.4%, and total lung capacity was 87 (±24). Post exercise oxygen saturation was normal in all patients (97 ± 1.5), and mean oxygen consumption max/kg was 18 ± 4.3 ml/kg/min (60.2 ± 15).
Mayberry et al. (2009) (n = 15)	USA Single Centre	Prospective observational study	Single episode of follow-up between 19-months and 8-years after injury	Univariate Analysis	SF-36 Physical Component score Employment Functional Status Overall health perception Pre-injury activity levels Co-morbidity Complications	Mean long-term MPQ *Pain Rating Index* was 6.7 (±2.1). SF-36 identified equivalent or better *health status* compared with references except for role limitations due to physical problems when compared with the general population. The operative fixation of BTI is associated with low long-term morbidity and pain, as well as HRQoL closely equivalent to the general population.
Leone et al. (2008) (n = 55)	France Single Centre	Prospective observational study	Follow up at 6-months and 1 year after injury	Univariate and Multivariate Analysis	Lung Function Tests Karnofsky Performance Status New York Heart Associated Classification St George Respiratory Questionnaire	71% (*n* = 39) had abnormal *Lung Function* *Physical function* was decreased in 70% (*n* = 38) 72% (*n* = 29) had a reduced 6-min walk distance Abnormal imaging was identified in 60% (*n* = 33) but this did not relate to lung function tests A ratio of arterial oxygen pressure to inspired oxygen fraction less than 200 at admission to ICU predicted abnormal lung function tests at 6 months.
Kerr-Valentic et al. (2003) (n = 40)	USA Single Centre	Prospective Observational study	4-months	Univariate and Multivariate Analysis	VAS pain score at 1,5,30,120 days post injury Short Form – 36 (30 days post injury) Total days out of work (120 days post injury)	Mean thoracic *pain* was 3.5 (±2.1) at 30 days and 1.0 (±1.4) at 120 days. When compared to the normative data, participants had higher *disability* at 30 days (*p* < 0.001) in all data sets excluding ‘emotional stability’, which indicated equivalent disability, and the perception of general health, where they were significantly less disabled (*p* < 0.001). The total mean days away from *work*/usual activity was 70 (±41). Days away from work were significantly lower in isolated BTI compared to those with extra-thoracic injury 51 (±39) vs. 91 (±33), *p* < 0.01
Mouton et al. (1997) (n = 23)	Switzerland Single Centre	Prospective Observational study	Single assessment, mean follow-up time 28 months	Univariate Analysis	Chest wall and shoulder girdle function Working capacity Sports activity Pain Chest wall deformity Morbidity	95% reported a 100% *working capacity* at assessment. 86% reported returning to pre-injury sporting activates without chest or shoulder girdle pain or dysfunction.
Beal & Oreskovich (1985) (n = 20)	USA Single Centre	Prospective observational study	Single assessment between 50 and 732-days post injury	Nil statistical analysis reported	Chest wall pain Chest wall deformity Exertional dyspnoea Employment status General Health Complications.	63.6% (*n* = 14) reported long term *morbidity*. Most common long-term problem = chest wall pain Pain prevented return to work in *n* = 3 participants
Landercasper et al. (1984) (n = 62)	USA Single Centre	Prospective observational study	Single assessment between 6 months and 12-years post injury	Nil statistical analysis reported	Dyspnoea Chest pain/tightness Employment history Lifestyle changes Chest x-ray Spirometry changes	38% (*n* = 12) had returned to full-time *employment* at point of follow-up 25% (n = 8) had subjective chest tightness 48% (*n* = 15) complained of chest wall *pain* 38% (*n* = 12) reported moderate to severe change in their overall level of activity.

### Physical impact of blunt thoracic injury

All of the included studies reported on the physical impact of BTI. These studies used different sample populations (i.e. varying injury severity, treatment requirements and presence of extra-thoracic injuries), outcome measures and measurement tools, methodology and follow-up timeframes. Notwithstanding these differences, these studies report that the most seriously injured patients with BTI are not fully recovered from the injuries they sustained after the specific study data collection period. The results of the studies in relation to the physical impact of BTI are presented below under the sub-themes of: (i) pain after BTI, (ii) physical function after BTI, (iii) post injury thoracic deformity, (iv) respiratory function after BTI.

#### Pain after blunt thoracic injury

On-going pain after BTI has been identified by 12 out of 15 studies included in this review and was identified as a key outcome measure [35–46].

##### Visual analogue scale

Of these studies, five measured pain using a Visual Analogue Scale (VAS) (Range: 0–10) [35, 36, 38, 39, 45]. Despite the limitations associated with VAS for pain assessment which includes potential for misinterpretation, risk of bias and a poor sensitivity to change; these studies identified on-going pain after BTI. Marasco et al. (2015) identified a mean VAS pain score of 2/10 (range: 0–10) at 6-months post injury and did not identify any statistically significant difference in pain scores reported by participants with isolated thoracic injury and those with associated extra-thoracic injuries at 12-months (mean 2 (range 0–5) vs. mean 2 (range 0–5) *p* = 0.35) and 24-months (mean 1 (range 0–4) vs. mean 0 (range 0–5) *p* = 0.5) after injury [39]. Similarly, Fabricant et al. reported mean VAS score of 3.5 at 1 month after injury despite high opiate usage amongst both participants with isolated and extra-thoracic injuries. When comparing the difference between thoracic pain and pain in other body parts, there was no statistically significant differences at any time point (day 1, 5, 30, 120) [36]. Furthermore, in a unique prospective observational study using trajectory modelling and VAS pain scoring, Daoust et al. identified pre-morbid smoking (Prevalence Ratio (PR): 1.8 (95% CI 1.3–2.6) *p* = 0.0009), the presence of two or more rib fractures (PR: 1.9 [95% CI 1.3–2.7], *p* = 0.0004) and initial oxygen saturations of less than 95% (PR: 1.7 [95% CI 1.1–2.6], *p* = 0.03) as statistically significant predictors of pain at 90 days after injury [45].

##### McGill pain questionnaire

Three studies measured patients’ sensory, affective and subjective experience of pain using the McGill Pain Questionnaire (MPQ) [36, 37, 43]. In the MPQ, an increased score indicates ‘worse’ outcomes and pain intensity. In a U.S. study (*n* = 203), at 2 months after injury the MPQ Pain Rating Index (PRI) and Present Pain Intensity (PPI) scales were a median of 1 (MPQ range: 0–5) and a mean of 10.6 ± 10.9 (PPI range: 0–44) respectively despite 24% of the sample remaining on opiate analgesic agents [36]. Similarly, Mayberry et al. reported a mean MPQ PRI of 6.7 ± 2.1 in their sample [43]. In this study, measurement used only one component from the MPQ and the follow-up was not undertaken at a pre-defined time point after injury which would likely introduce measurement bias into this study. Fabricant et al. and Gordy et al. reported that 59% (*n* = 110) and 22% (*n* = 35) respectively reported on-going chest pain and 76% (*n* = 142) and 53% (*n* = 86) respectively reported on-going disability associated with this pain at 2 months after injury [36, 37]. In the multivariate analysis showed enrollment MPQ PPI (undertaken during hospital admission) was an independent predictor of prolonged chest pain (Odds Ratio (OR): 1.6: 95% CI 1.1 to 2.3) but no specified injury characteristics predicted ongoing chest pain at 6 months after injury [36].

In a qualitative interview study using Interpretive Phenomenological Analysis (IPA), 14 participants were interviewed about their recovery after BTI between four and nine months post injury [44]. In this study, most participants identified learning to live with debilitating pain as a primary component of learning to cope with the injury itself.

#### Physical function

Physical function after BTI was quantified by nine studies included in this review using self-reporting methods [35–40, 42, 43, 47].

##### HRQoL component outcome measures

In both the SF-36 and SF-12 assessment tools, a higher score indicates ‘better’ outcomes, i.e. better HRQoL. Kerr-Valentic et al. measured the physical function score at one-month after BTI. This study (*n* = 40) compared the mean physical function score with the RAND reference group of chronically ill patients and found scores of 43.8 (± 29.6) vs. 70.6 (±27.4) for the reference group (*p* < 0.01) [35]. This highlights the level of comparative disability experienced by BTI patients at one-month after injury. In a prospective observational study (*n* = 203), Gordy et al. reported the mean physical function score at two-months (48.1), four-months (63.6) and six-months (70.4) but did not include standard deviation for these results and does not include the SF-36 summary component scores for physical and mental health in the univariate or multivariate statistical analysis [37].

At six-months after BTI, two studies by Marasco et al. reported no statistically significant differences in SF-12/SF-36 Physical Component Score (PCS) (39.7 (±12.6) vs. 37.3 (±12.3) *p* = 0.14) between those with isolated thoracic injury and those in the multiple trauma group and similarly (33.6 (±9.8) vs. 35.2 (±10.7) *p* = 0.65) between participants receiving operative rib fixation and those receiving conservative management [39, 47]. When the SF-12 PCS was compared with the Australian normative data, there were significant differences in the PCS with the Australian norms for physical function being significantly higher than the study population with BTI (Australian 2013 published norm: 48.9 (±10.2) *p* < 0.0001). Similarly, the outcomes reported at both 12 and 24 months were the same with reduced functional ability reported at each timeframe (PCS 12 months v.s. 24 months: 38.6 (±0.9) v.s. 38.9 (±1.0) p = NS) [39].

##### Other physical function outcome measures

One observational study compared long-term outcomes amongst BTI patients after operative fixation (*n* = 10) using two different rib fixation prosthesis (Synthes and Stratos prosthesis). In this study, the EORTC QLQ-C30 questionnaire was used to measure functional outcomes and quality of life despite the tool being developed for use with cancer patients without previous validation in a trauma population [38]. This study reported limited quantitative findings of functional outcomes in both patient groups but reported that the functional scale and symptoms scale outcomes were lower in the Stratos group – this was not statistically significant [38].

#### Post injury thoracic deformity

Four studies assessed participants for visible structural changes in the thoracic wall [40–42, 47]. The incidence of chest wall deformity was identified as 21.4% (*n* = 3) and 26.9% (*n* = 7) in two studies [40, 41]. In one study, 46% (*n* = 12) were unable to expand their chest more than 5 cm demonstrating significantly reduced respiratory function [40]. These studies link the impact of chest wall deformity with changes in respiratory function in the post injury recovery phase which is discussed below. Furthermore, these data are limited due to the single episode of follow-up undertaken between 50 days and 12 years after injury but identifies a relationship between thoracic deformity, respiratory function and HRQoL.

#### Respiratory function after blunt thoracic injury

Optimised respiratory function is understood to be a key variable in the prevention of respiratory complications after BTI. Three studies investigated respiratory function through follow-up chest radiology, physical examination, spirometry including carbon monoxide diffusion, smoking history and subjective and objective dyspnoea assessment [40, 41, 48]. The incidence of post injury dyspnoea was identified as 63% (*n* = 20) and 29% (*n* = 4) respectively at a single variable point of follow-up after discharge from hospital [40, 41]. Unfortunately, Beal & Oreskovich failed to present any specific data on pulmonary function tests preventing further inclusion of results in this review [41]. Similarly, Landercasper et al. reported only the incidence of respiratory abnormality with 57% (*n* = 12) of the sample producing abnormal spirometry results at a single variable point of follow-up after discharge from hospital. Furthermore, there was a 75% reduction in participant self-reported smoking in the post injury follow-up (although it is not clear whether this included both those who reduced daily smoking habits and those who quit completely). Interestingly, 27% (*n* = 4) had lower than normal lung volumes and 100% (*n* = 26) had abnormalities on chest X-ray consistent with pulmonary fibrosis [40]. Leone et al. identified only a partial pressure of oxygen/fraction of inspired oxygen (PaO2/FiO2) ratio of less than 200 on admission as a determinant of prolonged pulmonary complications in a predictive model. Pre-injury status, injury severity, ICU treatments and initial CT findings did not predict impaired pulmonary function [49].

In the qualitative interview study, both pain and difficulty in breathing were identified as a significant challenge that participants struggled to overcome. This important finding further highlights reduced respiratory function after BTI and the impact this has on physical function despite participants reporting that symptoms improved slightly each day [44].

### Psychological impact of blunt thoracic injury

Mental health squelae after BTI was assessed in three of the studies included in this review [37, 39, 47]. These studies have all used the Mental Component Score (MCS) of the SF-36 or SF-12 to measure the impact of psychological sequelae on HRQoL after BTI. Marasco et al. lead two studies that reported no statistically significant difference in the SF-12 MCS when comparing participants with isolated thoracic injury and those with associated extra-thoracic injuries at 6 months after injury (49.2 (SE 12.4) vs. 48.3 (SE 14.4) *p* = 0.61) and between participants who received operative rib fixation and those who were managed conservatively (45.1 (SE 13.8) vs. 45.2 (SE 9.3) *p* = 0.98) [39, 47].There was a statistically significant difference in SF-12 MCS when compared with the Australian norm: SF-12 MCS was significantly higher than the collective participants of this study (Australian 2013 published norms: 52.4 (SE 8.8) *p* < 0.0001) [39]. This study also identified that older patients (over 55 years) had significantly better SF-12 MCS scores (50.9 (SE 1.1) vs. 47.1 (SE 1.0) *p* = 0.01) and female participants had significantly lower SF-12 MCS than men (35.0 (SE 1.5) vs. 41.2 (SE 0.8) *p* < 0.001). There was no statistically significant difference in the outcomes of the SF-12 MCS at six-months, 12-months or 24-months after injury (48.8 (SE 0.9) vs. 49.3 (SE 0.9) vs. 48.9 (SE 1.0) *p* = 0.86) [39]. This study surmises that the better psychological outcomes in patients over 55 years was associated with a greater ability to accept physical disability in older age, although no evidence to support this was presented. Similarly, Gordy et al. presented the SF-36 mental health scores at two-months, four-months and six-months after injury (67.6, 72.1 and 73.3 respectively). Unfortunately, these results were not included in the univariate or multivariate analysis without clear reason and this limited the ability to interpret these findings further [37].

In the qualitative study, Claydon et al. identified a feeling of desperation amongst those recovering from BTI. These participants reported feeling like ‘life was on hold’ whilst waiting for their injuries to heal and symptoms to subside. It appears that these feelings were exacerbated by a sense of helplessness, like nothing could be done to help them. It is clear from these interviews that this impacted on the participants’ ability to regain some semblance of normality whist recovering from their injuries [44]. Although it was not explicitly explored in this study, it is likely that this would negatively impact on the QoL for those involved.

### Socio-economic impact of blunt thoracic injury

Six of the 15 studies reported outcomes relating to lost work days, functional ability to work and capacity to work [35, 39–43]. In these studies, assessment of capacity to return to work and duration of absence from work formed both an assessment of the socio-economic burden of BTI and as a method of further quantifying participant physical functional ability. Mouton et al. identified that 95% (*n* = 22) of participants could return to full pre-injury employment [42]. The sample in this study included patient with flail segments who had received surgical fixation of fracture. This may suggest that surgical fixation of rib fractures reduced the burden of injury considerably compared to those who have conservative management. Conversely, Marasco et al. reported that 30% of participants with BTI managed conservatively had not returned to pre-injury employment at two-years after injury [39]. Furthermore, Landercasper et al. found that only 34% (*n* = 12) of their sample with flail chest who did not receive surgical fixation returned to the same work after injury [40]. This study suggests that those who receive definitive treatment for fractures have improved outcomes and return to work earlier.

Post injury unemployment due to reduced capacity was measured through three studies as 14% (*n* = 2) [41], 33% (*n* = 9) [43] and 39% (*n* = 11) [40]. Returning to work part-time due to associated disability was reported as 11% (*n* = 3) [43] and 7% (n = 1) [41] of participants and a further 11% (n = 3) reported inability to work due to decreased functional ability after injury [43]. Furthermore, Kerr-Valentic et al. identified a mean loss of 70 work days (range: 29–111) due to BTI. In this study, those participants with isolated BTI returned to work faster than those with additional extra-thoracic injuries (51 ± 39 days vs. 91 ± 33 days, *p* < 0.01) respectively [35] (Additional file 1).

## Discussion

There is a strong evidence base relating to the outcomes reported by BTI victims during the acute post injury hospitalisation [50–53]. In contrast to this, the evidence available surrounding long-term outcome after BTI is inadequate due to issues in study methodology, inconsistent follow-up periods and the various outcome measures used in the included studies. Furthermore, in the general trauma population there is evidence suggesting that injury has a long-term impact on function state, mental health, HRQoL and return to a productive work life [26, 32, 54]. Patients with BTI present a further complexity in clinical practice because of the high level of underlying organ injury, associated pain and respiratory complications such as pneumonia [2, 52, 55, 56]. Although outcomes are described in the literature, these have not been previously attributed to specific BTI injury patterns [54].

Indicators of injury severity have been identified as a key predictor of negative long-term outcomes [39]. Flail chest itself has been identified as an indicator of severe injury to the thorax [36, 57]. Patients with flail chest were investigated in six studies [36, 38, 40–42, 47]. Of these, four studies reported using operative fixation of rib fracture for definitive management of flail chest [38, 42, 43, 47]. The remaining studies included in this review included participants with varying numbers of rib and sternal fractures (range: 1–16 thoracic fractures) suggesting a substantial difference in injury severity between participants and study populations [35–37, 39, 41]. This difference in the number of rib fractures requires careful consideration in the integration of the results of these studies to prevent bias and over estimation of the impact of the injury on functional status. Furthermore, four studies included in the review compared the long-term outcomes of patients with isolated thoracic injury with those who had associated extra-thoracic injuries [35, 39, 41, 43]. Whilst this provides a greater insight into the specific outcomes of these groups, there is added complexity in the assessment of injury severity amongst those with isolated thoracic injury and those with associated extra-thoracic injury [58]. This is likely to be a result of traditional methods of measuring injury severity being less responsive to patients with isolated BTI, potentially leading to under-estimation of morbidity and mortality.

The review also found inconsistent use of outcome measures in current studies. The measures that have been used are often not robust (e.g. 4 point Likert Scale) or do not have proven reliability and validity with physically injured patients (e.g. EORTC QLQ-c30). This finding is supported by Hoffmann et al. who highlighted the current limitations of using generic outcome measurements (e.g. SF-36) in the trauma population. This often results in health outcomes that are not comprehensively explored through the tools available [12]. This highlights the need for a trauma specific outcomes measure that is valid and reliable, specifically for trauma patients [12, 13, 59, 60].

The rich data reported by Claydon et al. from the qualitative interview study demonstrates how qualitative research can add to the knowledge surrounding outcome measures in a specific population [44]. This highlights that qualitative research has an important role and can provide a unique and critical contribution to health outcomes research [61]. The literature included in this review was predominately quantitative in nature. Despite this, there is a general agreement between the findings in both the quantitative studies and the one qualitative study. Further qualitative studies will allow a greater understanding of the patient experience of recovering after BTI and exploration of which outcomes are meaningful of patients.

There was inconsistency in the follow-up timeframes used by studies. Nine studies measured outcomes at a single time point which varied from 50 days to 12 years after injury [38, 41–43, 45–49], whilst four studies used longitudinal follow-up over a predefined timeframe [35–37, 39]. Apart from the obvious challenges associated with synthesising the data from studies undertaken over differing timeframes, Gabbe et al. highlights the importance of careful consideration of timeframes for health-outcomes studies in trauma research [27]. Furthermore, there was variation in recovery rates for certain sub-groups highlighting the need for reflection on both the outcome measures of interest and the specific population being studied [27]. This shows how a more standardised approach to trauma health outcomes research could result in a more robust repository of knowledge in the future.

Although the extended interval between injury and return to work is indicated in these studies, they have not comprehensively measured the socio-economic impact of BTI [62, 63]. There are potentially confounding variables that have not been measured that may influence a patient’s return to employment including: socio-economic status, physical demands of role, duration at present job, associated work-related benefits, role flexibility, job satisfaction and individual’s motivation to work [62, 64]. Similarly, it is important to consider the impact of differing levels of social support and compensation/legal practices seen in the countries where the research was conducted, as this could influence a participant’s motivation to return after injury [65–69]. Furthermore, variables including pre-injury health status, alcohol and illicit substance misuse behaviours are also likely to impact on the socio-economic impact of BTI [62]. These suggest that further research is required to fully understand the variables contributing to the socio-economic burden of BTI.

### Limitations

In this narrative review, every effort was made to undertake comprehensive searches using systematic and thorough methods. All papers that were identified and available were retrieved and assessed against the eligibility criteria which included both qualitative and quantitative studies. Since there was obvious hetrogeniety in the variables, outcome measures and patient samples used in the studies included, it was not within the scope of this review to undertake a synthesised quantitative analysis. Despite this, it offers a topical investigation of current evidence surrounding the long-term outcomes for patients with BTI that has hitherto been missing from the international literature.

## Conclusion

The review has highlighted that more robust research is necessary to isolate the true effect of BTI on the individuals involved and their families. Developing a greater understanding of the negative impact of BTI will benefit both the patient, through easier access to interventions, and could also decrease the associated implications on the wider society. The evidence included in this review identified the need for clinical staff to consider the long-term outcomes of trauma patients when planning care and assessing ongoing care needs beyond the acute hospital admission. This transition from measuring traditional trauma outcomes to a more functional assessment is essential to the ongoing progression of the major trauma system throughout high-income countries. A reliable method for assessing functional outcomes in trauma is key to the measurement of the effectiveness of trauma care. This could lead to the development of a more responsive and predictive care pathway that aims to identify risk of long-term sequelae and reduce the persistent disability and burden of BTI.

## Additional file

Additional file 1: Table S1 Results of Included studies. (DOCX 32 kb)

## Abbreviations

BTI: Blunt Thoracic Injury
CASP: Critical Appraisal Skills Programme
CINAHL: Cumulative Index to Nursing and Allied Health
CT: Computerised Tomography
ED: Emergency Department
EORTC: European Organisation for Research and Treatment of Cancer
ETI: Extra-Thoracic Injury
HCP: Health Care Professional
HEE: Health Education England
HRQoL: Health-Related Quality of Life
ICU: Intensive Care Unit
IPA: Interpretative Phenomenological Analysis
ISS: Injury Severity Score
MCS: Mental Component Score
MPQ: McGill Pain Questionnaire
NIHR: National Institute for Health Research
NIV: Non-Invasive Ventilation
PCS: Physical Component Score
PPI: Present Pain Index
PRI: Pain Rating Index
PRISMA: Preferred Reporting Items for Systematic Reviews and Meta-Analysis
PROMs: Patient Reported Outcome Measures
QoL: Quality of Life
RCT: Randomised Controlled Trial
SD: Standard Deviation
SE: Standard Error
SF-12: Short Form – 12 Health questionnaire
SF-36: Short Form – 36 Health questionnaire
TOP: Trauma Outcome Profile
UK: United Kingdom
VAS: Visual Analogue Scale
