# Supplementary material for: The long-term outcomes and health-related quality of life of patients following blunt thoracic injury: a narrative literature review
Source: Scand J Trauma Resusc Emerg Med. 2018 Aug 17;26:67. doi: 10.1186/s13049-018-0535-9 (PMC6098638; doi:10.1186/s13049-018-0535-9)
Supplement: Supplementary file 1 — Table S1 Results of Included studies. (DOCX 32 kb) [file 13049_2018_535_MOESM1_ESM.docx]

Table S1 Results of Included studies

|  | **Physical Impact** | | | | | **Psychological Impact** | **Socio-economic Impact** |
| --- | --- | --- | --- | --- | --- | --- | --- |
| **Author/**  **Population** | **Pain** | **Pulmonary Function** | **Physical Function** | **Chest wall deformity** | **Complications** | **Mental health** | **Employment** |
| **Marasco et al. (2015)**  (n=397)  Isolated rib fractures (n=216)  chest injury + extra-thoracic injury (n=181) | Mean VAS pain score  (range: 0-10)  **Univariate analysis:** (Thoracic vs. Extra-Thoracic)  **6/12:**  2 (0-5) vs. 2 (0-5) p=0.35  **12/12:**  0 (0-5) vs. 2 (0-5) p=0.09  **24/12:**  1 (0-4) vs. 0 (0-5) p=0.50  **Multivariate analysis:**  **Female**: 3.5 (0.3)  **Male**: 2.2 (0.2)  p=0.002  **>55** **years**: 2.8 (0.2)  **≤ 55** **years**: 2.9 (0.2) p=0.75  **6/12**: 3.1 (0.2)  **12/12**: 2.9 (0.2)  **24/12**: 2.6 (0.2)  p=0.03 | Not measured or reported | SF-12 PCS  **Univariate analysis:**  (Thoracic vs. Extra-Thoracic)  **6/12:**  39.7 (12.6) vs. 37.3 (12.3) p=0.14  **12/12:**  41.4 (12.4) vs. 39.4 (13.8) p=0.26  **24/12:**  42.5 (12.2) vs. 39.2 (14.0) p=0.11  **Multivariate analysis:**  **Female:** 49.1 (1.4)  **Male:** 48.8  p=0.84  **>55 years**: 37.9 (1.1)  **≤55 years**: 38.3 (1.1)  p=0.81  **6/12**: 36.9 (0.9)  **12/12**: 38.6 (0.9)  **24/12**: 38.9 (1.0)  p=0.01 | Not measured or reported | Not measured or reported | SF-12 MCS  **Univariate analysis:**  (Thoracic vs. Extra-Thoracic)  **6/12:**  49.2 (12.4) vs. 48.3 (14.4) p=0.61  **12/12:**  49.2 (11.1) vs. 49.2 (12.8) p=0.99  **24/12:**  49.7 (11.2) vs. 47.9 (12.5) p=0.32  **Multivariate analysis:**  Female: 35.0 (1.5)  Male: 41.2 (0.8)  P=<0.001  >55 years: 50.9 (1.1)  ≤55 years: 47.1 (1.0)  p=0.01  6/12: 48.8 (0.9)  12/12: 49.3 (0.9)  24/12: 48.9 (1.0)  p=0.86 | **Pre-injury:**  2% not working  86% worked FT  12% worked PT  **6/12:**  59% had returned to work  94% of these returned to pre-injury employment  Thoracic vs. extra-thoracic:  63% (n=59) vs. 51% (n=47) p=0.16  Results at **12/12** and **24/12** were unchanged from 6/12. |
| **Gordy et al. (2014)**  (n=203)  Isolated chest (n=29)  Extra-thoracic (n=114) | MPQ – PPI and PRI  **At enrolment:**  PPI: median 3 (range: 0-5)  PRI: mean 28±16 (range 0-70)  22% reported chronic pain at 6/12  Total sample:  **Univariate analysis:**  Extra-thoracic injury and Enrolment PPI associated with chronic pain (p=0.03 and p=0.003 respectively)  **Multivariate analysis:**  Enrolment PPI predicted chronic pain at 6/12 (OR: 1.44, 95% CI 1.01-2.07)  Isolated chest group:  **Univariate analysis:**  Nil associated variables  **Multivariate analysis:**  Nil predictive variables | Not measured or reported | SF-36 Health Survey PCS:  Total Sample:  **2/12**: 14.8  **4/12**: 37.5  **6/12**: 48.1  Isolated Chest Group:  **2/12:** 20.4  **4/12:** 47.0  **6/12:** 55.6  SF-36 Not included in any univariate or Multivariate analysis in this publication. | Not measured or reported | Not measured or reported | SF-36 Health Survey MCS:  Total Sample:  **2/12**: 67.6  **4/12**: 72.1  **6/12:** 73.3  Isolated Chest Group:  **2/12**: 67.6  **4/12:** 73.2  **6/12:** 73.7  SF-36 Not included in any univariate or Multivariate analysis in this publication. | Not measured or reported |
| **Marasco et al. (2013)**  (n=46)  Group one: operative (n=23)  Group two: non-operative (n=23) | Bodily Pain (SF-36)  **Group 1:** Operative  42.2±9.4  **Group 2:** Non-operative  37.9±11.0  p=0.22 | No-significant difference between Spirometry results at 3/12 after injury | SF-36 PCS at 6/12:  **Group 1:** Operative  PCS: 33.6±9.8  **Group 2:** Non-operative  PCS: 35.2±10.7  p=0.65 | There was no significant difference in the degree of healing and residual deformity between operative and non-operative groups. | Not measured or reported | SF-36 MCS at 6/12:  **Group 1**: Operative  MCS: 45.1±13.8  **Group 2**: Non-operative  MCS: 45.2±9.2  p=0.98 | Not measured or Reported |
| **Daoust et al. (2013)**  (n=734) | 46.6% reported low pain at 2/52 and low pain at 3/12  35.2% reported Moderate pain at 2/52 and low pain at 3/12  18.2% reported moderate pain at 2/52 and moderate pain at 3/12  Trajectory Modelling:  (Prevalence ratio (PR))  Smoker: PR 1.8 (1.3-2.6) p=0.0009  >2 rib #: PR 1.9 (1.3-2.7) p=0.0004  SpO2 <95% at admission: PR 1.7 (1.1-2.6) p=0.03 | Not measured or reported | Not measured or Reported | Not measured or reported | Not measured or reported | Not measured or reported | Not measured or reported |
| **Bille et al. (2013)**  (n=10)  Stratos prosthesis (n=4)  Synthes prosthesis (n=6) | VAS Pain Score (range: 0-10)  Median pain score: 0 (0-8)  Pain Score 0: n=7  Pain Score 1: n=1  Pain Score 4: n=1  Pain Score 8: n=1  Impact of pain:  Quite a lot (n= 2)  Little bit (n=3)  No impact (n=5)  n=2 continue to use regular opiate analgesics at follow-up | Not measured or reported | QLQ-C30 Functional and Symptom Scale  **Limitation on Physical Activity:**  Substantial: n=3  Mild: n=3  No limitation: n=4  **Stratos group:**  (Median Value)  Functional Scale: 53.5 (range: 20-100)  Symptom Scale: 39.5 (range: 0-100)  **Synth Group:**  (Median Value)  Functional Scale: 93 (range: 70-93)  Symptom Scale: 2.35 (range: 0-33)  Functional Scale value 93 = higher ‘better’ level of Physical Function and value of 39.5 in the Symptom Scale indicated higher ‘worse’ level of symptoms. | n=0 due to the surgical fixation of malunion of rib fractures | n=1 – contra-lateral pleural effusion requiring post-operative drainage. | Not measured or reported | Mean return to work: 2 months  Nil further specific data provided |
| **Fabricant et al. (2013**)  (n=203)  (n=111) Isolated rib fractures  (n=92) Chest injury + extra-thoracic injury | Total Sample:  59% (n=110) reported prolonged pain at 2/12 after injury.  Isolated Chest group:  64% (n=67) reported prolonged pain at 2/12 after injury  Enrolment MPQ PPI and PRI:  PPI: median 3 (range: 0-5)  PRI: mean 28 ±16 (range: 0-70)  2/12 MPQ PPI and PRI  PPI: median 1 (range: 0-5)  PRI: mean 10.6 ±10.9 (range: 0-44)  **Multivariate analysis:**  Initial MPQ PPI was predictive of prolonged pain (OR: 1.8, 95% CI 1.4-2.5) at 2/12 after injury | Not measured or reported | Total Sample:  76% (n=142) reported prolonged disability at 2/12 after injury.  Isolated Chest group:  66% (n=69) reported prolonged pain at 2/12 after injury  **Multivariate analysis:**  Initial MPQ PPI was predictive of prolonged disability (OR: 2.2, 95% CI 1.5-3.4) and Presence of Extra-thoracic injury was predictive of prolonged disability at 2/12 after injury (OR: 5.9, 95% CI, 1.4-29 | Not measured or reported | Not measured or reported | Not measured or reported | Not measured or reported |
| **Shelat et al. (2012)**  (n=102) | 22.5% (n=23) complained of chronic persistent pain  26% (n=6) with chronic pain reported using regular analgesics  35% (n=8) complained of impaired work life  13% (n=3) complained of impaired personal quality of life | Not measured or reported | Not measured or reported | Not measured or reported | Not measured or reported | Not measured or reported | Not measured or reported |
| **Amital et al. (2009)**  (n=13) | Not measured or reported | Mean FEV1: 8.1±15.3%  Vital Capacity: 101±14%  CO diffusion Capacity: 87±24  Post exercise SpO2: 97%±1.5% | Not measured or reported | Not measured or reported | Not measured or reported | Not measured or reported | Not measured or reported |
| **Mayberry et al. (2009)**  (n=15) | MPQ PRI:  Mean 6.7 ±2.1 at follow-up  Operative fixation of rib fractures was associated with reduced pain at follow-up. | Not measured or reported | SF-36 PCS  Identified equivalent or better health status compared with the Medical Outcomes Study reference group except for role limitation due to physical problems when compared with the general population. | Not measured or reported | After operative fixation of rib fractures, there were two episodes of deep wound infection and 13% reported fixation failure (n=6) | Not measured or reported.  The SF-36 PCS was used in isolation in this study. | 33% (n=9) reported being unable to return to work after injury  11% of the population reported returning to work on a PT basis due to limited functional ability after injury |
| **Leone et al. (2008)**  (n=55) | Not measured or reported | Lung Function Test abnormality was identified in 71% (n=39)  A ratio of PaO2 to FiO2 less than 200 at admission to ICU predicted abnormal lung function at 6/12 | Physical function was decreased in 70% (n=38)  6-minute walk distance was reduced in 72% (n=29) | Not measured or reported | Not measured or reported | Not measured or reported | Not measured or reported |
| **Kerr-Valentic et al. (2003)**  (n=40) | VAS pain scale:  Mean pain:  **1/12**: 3.5 ±2.1  **4/12**: 1.0 ±1.4  Isolated chest vs. extra-thoracic pain:  **1/12:** 3.3 ±2.7 vs. 2.3 ±2.5 (non-Significant)  **4/12**: 1.0 ±1.6 vs. 2.4 ±2.7 (non-significant) | Not measured or reported | SF-36 PCS (comparison with the RAND Medical Outcomes Study – Chronic Illness)  Total sample vs. RAND reference group:  Physical Function:  43.8 ±29.6 vs. 70.6 ±27.4 p<0.01  Physical Limitation:  8.6 ±22.5 vs. 53 ±40.8 p<0.01  Chest injury patients were more disabled at 30 days (p<0.001) in all categories except emotional stability, where they showed equivalent disability and in perception of general health they were less disabled (p<0.001) | Not measured or reported | Not measured or reported | This study used the PCS of the SF-36 only  Not measured or reported | Patient with isolated chest injuries returned to work/usual activity at a mean of 51 ±39 days compared with 91 ±33 days in patients with associated extra-thoracic injuries (p<0.001) |
| **Mouton et al. (1997)**  (n=23) | Prolonged pain experienced by 24% of sample (n=5)  Reports of shoulder gridle pain was most commonly reported. | Not measured or reported | 86% reported being able to return to a pre-injury level of physical activity including sports. | No chest wall deformity reported -probably associated with the | Not measured or reported | Not measured or reported | 95% reported ability to return to pre-injury work. |
| **Beal and Oreskovich (1985)**  (n=20) | N=5 reported substantial chronic chest pain at follow up | Pulmonary function test undertaken in follow up but results not explicitly reported in the publication  n=6 reported dyspnoea at follow up | Not measure or reported | Chest wall deformity in n=3  In one, 50% thoracic volume loss on CT due to deformity. | n=1 pleural effusion  n=1 neuroma  n=1 long term mechanical ventilation requirements | Not measured or reported | n=3 exertional chest pain prevented return to work post injury |
| **Landercasper et al. (1984)**  (n=62) | 25% (n=8) reported prolonged chest tightness.  49% (n=15) reported prolonged chest pain | Subjective dyspnoea was reported by 63% (n=20). Of n=15 pre-injury smokers, n=10 had stopped post injury  n=26 had chest XR and 100% had evidence of pleural thickening/fibrosis  46% (n=12) unable to expand chest more than 5cm  Abnormal spirometry in n=12  Mild objective dyspnoea diagnosed in n=10  Moderate dyspnoea diagnosed in n=4 | 37% (n=12) reported moderate to severe changes in level of physical function.  72% (n=23) reported slight changes to lifestyle | 26.9% (n=7) reported chest wall deformity at follow-up | Not measured or reported | Not measured or reported | Not working at time of injury (n=4)  43% (n=12) returned to previous full time employment.  7% reported changing job due to injury (n=2)  11% (n=3) returned to work but on a part time basis |
| Qualitative: | Results: | | | | | | |
| **Claydon et al. (2017)**  (n=14) | - *Theme 1: Struggling with breathing and pain:*    - Patient described struggling with breathing and chest pain that was initially very sever.   - ‘It takes your breath away’ Participants felt scared that they may not survive the injuries and recovery process.   - Patient generally reported that symptoms improved with time but the sensation of being ‘out of puff’ extended for a substantial period of time. - *Theme 2: Life on hold:*   - Healing was considered a natural process which people couldn’t influence, creating frustration whilst waiting for injuries to heal Many believed they would never fully recover and accepted limitations - *Theme 3: Lucky to be alive*:   - All participants expressed a sense of feeling lucky to be alive. The seriousness of injury prompted a change in attitude to make the most of life. | | | | | | |
